# Reply of the Graduating Class to the Valedictories of Professors Allen and Smith

**Published:** 1854-04

**Authors:** G. Watt


### *Reply of the Graduating Class to the Valedictories of Professors* ALLEN *and* SMITH. *By* DR. G. WATT.

TO THE FACULTY OF THE OHIO COLLEGE OF DENTAL SURGERY—GENTLEMEN—The present moment is to us one of peculiar interest. It is a period in which strong ties are severed—strong feelings aroused. The social ties binding us together as classmates are now to be severed, as each selects his field of labor.

But other ties are rent. For months we have been your pupils, and pleasant to us has been the relation, but, by this evening's acts, you have declared us your pupils no longer. We have looked up with longing eyes to the platform of professional distinction occupied by you — now you have lifted us up, and set us beside you.

If we may be allowed to believe that this act is not a mere act of clemency, but the result of your deliberate judgment, aided and assisted by the examining committee, it would be veriest affectation of modesty to say we do not feel flattered. But when we reflect that your own integrity, your sense of justice, your responsibility to the public, and your *jealousy* for the honor of your profession, would all influence your decisions, we are proud of the position we now occupy.

This epoch in our lives is called our "commencement." It is the commencement of our professional career—the commencement of our life's struggle in the cause of health and humanity—the commencement of our professional battle with empiricism, in all of its forms, and under all its disguises. With progress for our watchword, *truth* for our emblem, and science for our standard-bearer, may we press on, assured that, in time, the victory will be ours.

Though always anticipated with longing eye, yet this period is never passed without regret.

Our relation to society being changed, new duties arise, new difficulties are to be encountered, new trials met, and new obstacles overcome.

The young soldier who, from a safe position, views the distant battle, knowing that in the next he will be a participant, is by no means a careless spectator. Thus have we looked on the trials and struggles of professional life. But being now "enlisted for the war," our *commissions* "signed and delivered," how infinitely more vivid become our views of the responsibilities involved. Feeling these responsibilities, and looking forward to the trials and difficulties, especially those to be met at the very outset, 'twere human nature to shrink from the contest. But, encouraged by your own success, influenced by your example, guided by your counsel, qualified by your instruction, and indorsed by your judgment, we hope to press on, resolved not to "do or die," but to *do.*

As we have looked forward to this occasion, so will we look back to it. The wanderer on the wide world, through youth, through manhood, and when his locks are gray, looks back, in memory, to the parental roof, and cherishes the recollectious of his childhood's home. And there is ever one scene which looms up, like the blue mountain-top, far above its fellows. This one scene, in all its circumstances, is impressed on every page of his memory's book. One day is sacred and dear above all days spent in that home. That scene is the parting scene—that day is the one in which the parental mansion ceased to be his home—the day in which he cast himself on the world, assuming the duties and responsibilities of manhood. So will our minds turn instinctively to this evening and its scenes—so will we remember the closing period of our collegiate, and the "commencement" of our professional life; and as the wanderer clings to and cherishes the parental gifts—the parting tokens—so will we honor and prize this,* and especially this†—the parting tokens of our "Alma Mater."

But, though our pupilage is terminated, still we are students. Vast fields of professional science lie open, inviting us to cultivate them; nay, much lies not only unoccupied, but unexplored, awaiting the visit of the hardy adventurer.

---

* The Diploma. † The Holy Bible.

There is then room for the fullest exercise of that professional ambition, which ever aims at the advancement of our profession for the good of our race.

May the progress of the past be our encouragement for the future; and as you and your brethren have borne your part in that which is past, may we hope to join with and assist you in that which is future. May we—may our profession—labor hand in hand, some cultivating principles already planted till they grow up to perfection—others exploring new fields and developing new principles, each pursuing that for which nature and the circumstances of life have fitted him—all aiming at that mutual improvement, which alone is capable of elevating the profession. While thus striving, may we cultivate a laudable spirit of emulation, a spirit of generous rivalry; but may we never mar the interests of our cause by professional jealousy, nor cripple its energies by professional selfishness.

In our intercourse with professional brethren, may we ever obey the spirit of that divine injunction, "Freely ye have received, freely give."

In the name of the class, I bid you farewell.

